# Development of a Low-Cost Markerless Optical Motion Capture System for Gait Analysis and Anthropometric Parameter Quantification

**DOI:** 10.3390/s24113371

**Published:** 2024-05-24

**Authors:** Laura Alejandra Espitia-Mora, Manuel Andrés Vélez-Guerrero, Mauro Callejas-Cuervo

**Affiliations:** Software Research Group, Universidad Pedagógica y Tecnológica de Colombia, Tunja 150002, Colombia; laura.espitia01@uptc.edu.co (L.A.E.-M.); manuel.velez@uptc.edu.co (M.A.V.-G.)

**Keywords:** motion capture, optical analysis, depth sensors, artificial intelligence, computer vision, RealSense, lower limbs, rehabilitation, sports, entertainment

## Abstract

Technological advancements have expanded the range of methods for capturing human body motion, including solutions involving inertial sensors (IMUs) and optical alternatives. However, the rising complexity and costs associated with commercial solutions have prompted the exploration of more cost-effective alternatives. This paper presents a markerless optical motion capture system using a RealSense depth camera and intelligent computer vision algorithms. It facilitates precise posture assessment, the real-time calculation of joint angles, and acquisition of subject-specific anthropometric data for gait analysis. The proposed system stands out for its simplicity and affordability in comparison to complex commercial solutions. The gathered data are stored in comma-separated value (CSV) files, simplifying subsequent analysis and data mining. Preliminary tests, conducted in controlled laboratory environments and employing a commercial MEMS-IMU system as a reference, revealed a maximum relative error of 7.6% in anthropometric measurements, with a maximum absolute error of 4.67 cm at average height. Stride length measurements showed a maximum relative error of 11.2%. Static joint angle tests had a maximum average error of 10.2%, while dynamic joint angle tests showed a maximum average error of 9.06%. The proposed optical system offers sufficient accuracy for potential application in areas such as rehabilitation, sports analysis, and entertainment.

## 1. Introduction

In recent decades, the study of human movement has experienced a significant transformation, driven by advancements in various technologies and techniques [1]. These innovations have found applications across multiple fields, including biomechanics, sports, medicine, computer animation, and the entertainment industry [2]. One of the primary driving factors behind this transformation has been the use of multiple cameras, infrared systems, acceleration sensors, and computer vision techniques, collectively revolutionizing the precision and adaptability of motion capture techniques [3,4]. This technological progress has fundamentally reshaped researchers’ understanding and application of human movement dynamics. This paper focuses on developing a motion capture system that seamlessly melds accessible optical technologies with state-of-the-art intelligent algorithms for optical data processing. The primary objective of this system is to streamline the real-time acquisition, analysis, and interpretation of human motion, representing a monumental leap in both accuracy and efficiency.

### 1.1. Motion Capture Systems and Applications

Motion capture technology, commonly known as MOCAP, has revolutionized numerous industries and fields of study by providing an indispensable tool for obtaining precise real-time or pre-recorded data on the motion and positioning of objects or subjects. Subsequently, computer-aided systems analyze these data.

In motion capture, there are two primary approaches: optical systems and inertial systems. Optical systems use specialized cameras and, in some cases, markers to record motion from multiple angles, thus creating an accurate three-dimensional representation of the captured motion [5,6,7]. In contrast, inertial systems rely on acceleration sensors and gyroscopes to directly record motion on the body or the target object. In research and development, the use of various sensor types and intelligent processing techniques has significantly improved the accuracy and versatility of motion capture, shaping the understanding of human motion knowledge.

These approaches offer distinct advantages and applications, such as in biomechanics, animation, and medical research. In the medical field, there is an increasing demand for tools that enable continuous monitoring to gather precise information about specific anatomical areas. This capability enhances the understanding of movement disorders and the development of customized medical devices. However, many of these devices often suffer from common limitations, including high costs, the need for specific installation locations, and challenges related to portability.

Moreover, in many industries, motion capture plays a key role, including film, animation, and video games [8], where it is employed to create lifelike animations and enhance visual effects. Additionally, fields such as biomechanics and sports analysis derive significant benefits from this technology, empowering researchers and professionals to assess and improve human movement, evaluate athlete performance, and proactively prevent injuries.

### 1.2. Proposal and Organization

This paper centers on the design, development, and preliminary validation of an affordable and user-friendly, markerless, optical motion capture system tailored for human gait analysis and the quantification of some anthropometric parameters. This system combines a depth camera with the open source MediaPipe (version 0.10.2) pose estimator. The objective of this implementation is to create a robust working prototype for evaluating both its performance and the accuracy of the collected data, thus preparing it for further analysis within the context of human gait.

The article’s structure is as follows. Section 2 provides a review of the state of the art, focusing on low-cost portable systems, motion capture system applications in health and rehabilitation, sensor usage, processing algorithms, and current trends in human motion capture. Section 3 details the materials and methodology utilized in the research, including the proposed design and implementation of the motion capture prototype. Section 4 presents the primary results obtained from evaluating the described system in a controlled environment. Section 5 discusses the obtained results. Finally, Section 6 summarizes the conclusions drawn from the study and outlines future research directions.

## 2. Current Advances in Motion Capture Technology

The growing demand for innovative and affordable solutions in low-cost wearable devices has driven technological advances that play a crucial role in acquiring accurate data in fields, such as healthcare, rehabilitation, biomechanics, and sports analysis. This section provides an overview of the current state of these technologies, emphasizing their influence on medical research, rehabilitation, biomechanical analysis, and emerging trends that are reshaping the comprehension and utilization of movement-related knowledge [9].

### 2.1. Health and Rehabilitation Applications

In the healthcare and rehabilitation fields, challenges have arisen due to the absence of guidelines and algorithms for measuring gait metrics, along with the high costs associated with commercial motion capture systems. To address this need, research has explored the use of low-cost, highly portable handheld devices.

Various technologies have been developed for capturing human motion. These include the use of a Kinect system for health exercises [10], high-precision optical systems [11], fast-processing markerless systems [12], and commercial systems like *KinaTrax* for improved accuracy [13]. Additionally, videography-based systems utilizing computer vision and deep learning have been developed for 3D gait analysis [14,15,16], demonstrating results comparable to marker-based systems. Nevertheless, it is imperative to exercise caution owing to the potential for errors in the analytical methods employed.

The integration of both visual and biomedical data, such as electromyography (EMG), inertial measurement units (IMUs) data, and data from other medical instruments, enhances the performance of prostheses. A highly accurate EMG-based functional range signal (EFRS) system, especially in the classification of common amputee environments, underscores its effectiveness in controlling lower limb prostheses [17]. Additionally, a methodology using an IMU and a depth sensor demonstrates the precise prediction of heel and toe contact events, which can improve the usability and safety of lower limb assistive devices [18].

Other studies assess gait parameters in patients with neurological diseases by employing a single RGB-D sensor, complemented by wearable sensors to collect kinematic, kinetic, and biochemical data [19,20]. This approach helps rectify inaccuracies in sensor data, enhancing the precision of monitoring and evaluating gait and rehabilitation-related parameters in patients with compromised mobility conditions.

### 2.2. Processing Algorithms

In motion capture and data analytics for health and rehabilitation, the fusion of sensors and processing algorithms is relevant to obtain accurate data. Optical sensors, such as Intel’s RealSense optical sensor mentioned in [21], are essential for object detection and information transfer, but often face challenges related to noise and gaps in their performance. To address these issues, several solutions have been proposed, including the *gradientEMM* method and specialized deep neural networks [22].

Several techniques have been introduced for the 3D reconstruction of human models from depth cameras, including the *kinectFusion* technique [23]. Additionally, other studies have showcased its descriptive capabilities by implementing 2D skeletons, demonstrating competitiveness in classifying skeletons within 3D models [24]. Furthermore, the use of the RealSense T265 sensor, which employs VI-SLAM for precise position and velocity estimation, has been documented [25], while other research has highlighted this sensor’s capacity to capture gait data through point cloud generation [26]. Finally, similar sensors, like the RealSense D415, have also been regarded as practical and cost-effective choices for facial anthropometric measurements with portability requirements [27].

Computer vision has proven highly effective in capturing human motion data, utilizing various neural networks and deep learning techniques to extract information about skeletal joints from videos of individuals engaged in diverse locomotor activities [7,14,28,29,30,31]. In addition to computer vision, attention has been directed toward other complementary approaches and algorithms, including the K-nearest neighbor algorithm applied in human action recognition [32,33,34]. Furthermore, significant focus has been placed on exploring pose estimation algorithms, which have demonstrated remarkable accuracy in sports motion capture [35]. These advancements in motion feature extraction and analysis hold substantial potential, particularly in fields such as biomechanics and sports research, where precise motion capture and evaluation are pivotal for enhancing comprehension and enhancing human performance.

### 2.3. Trends in Human Motion Capture

Human motion capture technology has undergone remarkable advancements, as detailed in the study [9], which specifically focuses on lower limb research. 

This literature review analyzing research papers published between 2018 and 2022 reveals a diverse array of technologies in use: 41% employ optical sensors, 24% utilize MEMS sensors, 14% rely on radar sensors, and 21% combine MEMS and optical sensor technologies. These findings underscore the diverse technological approaches within human motion capture, focusing on applications related to the lower limbs.

Significant progress has been achieved in processing and control techniques, particularly in the domain of computer vision, where deep learning algorithms and data science-based predictive models are in constant evolution. Moreover, videos have become a prevalent tool for capturing and processing information due to their versatility in representing events and situations [14,19,28,36,37,38]. These examples demonstrate how the integration of videos with computer vision methods and 2D/3D models enables a comprehensive analysis of human motion.

Furthermore, there is a growing interest in versatile intelligent algorithms that do not rely solely on neural networks, as they offer efficiency and significant enhancements in anthropometric measurements. MEMS-based data fusion algorithms, such as Kalman filtering, are commonly employed to enhance measurement accuracy and mitigate computational errors. 

Additionally, approaches like smoothing techniques and robust algorithms designed for challenging conditions, unexpected noise, and segmentation play a pivotal role. These highlights underscore the importance of algorithms in optimizing and enhancing the quality of sensor-captured data [1,7,32,39,40,41,42,43].

The development of these filtering and optimization algorithms has been instrumental in enhancing the accuracy and reliability of inertial data. For instance, the Kalman filter has been extended to provide more precise updates during dynamic motion. Variants like the complementary Kalman filter have also been devised, serving the dual purpose of fusing data and effectively mitigating computational errors.

Lastly, another critical approach involves the application of parameter optimization algorithms customized for specific applications. These algorithms may encompass smoothing techniques to minimize undesired fluctuations and robust algorithms to address challenging conditions and unexpected noise in the collected data [44,45,46]. 

## 3. Materials and Methods

This section provides the data collection process, including the instruments and tools utilized, alongside the analytical techniques employed for data processing. Additionally, it outlines the experimental procedures and addresses how the results in developing the proof of concept for this system will be discussed.

### 3.1. Method

The research employs a rigorous quantitative methodology, which places a strong emphasis on the acquisition and processing of information using numerical magnitudes and formal techniques. This approach enables precise and concrete analyses, ensuring the reliability of the findings. The research team has chosen to adopt the hypothetico-deductive method as their guiding principle, commencing with the hypothesis that the optical system for gait analysis yields high-quality data. This hypothesis was rigorously tested through empirical validation in the study.

This strategic blend of quantitative and deductive approaches established a robust framework for investigating and advancing the proposed motion capture system. Table 1 below offers a concise overview of the key stages in the research approach. 

These stages have been meticulously designed to ensure a coherent development aligned with the research objectives. The thorough planning and execution of these stages ultimately guarantee the acquisition of accurate and dependable results.

### 3.2. Materials

Table 2 below details the materials implemented in this study, clearly differentiating between the hardware (H) used in the computer infrastructure and the software (S) used for data development and analysis.

The system primarily relies on an Intel RealSense D435 depth camera, known for its compactness and portability. It has a range of up to 10 m and a speed of 90 FPS. Intelligent processing algorithms, developed in Python, were used to analyze the captured data and extract relevant information. These technologies work together to provide precise and dependable results, particularly in optical systems.

In terms of software resources, the core component was created using the MediaPipe pose estimator. This tool simplifies the development and execution of machine vision applications, offering customizable solutions compatible with various platforms. Its effectiveness and adaptability make it valuable for creating and deploying machine vision applications, even on less powerful hardware.

Lastly, to establish a visual reference framework, ArUco synthetic markers were integrated. This visual information processing library enables the recognition of markers of different sizes and configurations. This functionality allows for marker recognition, even when rotated or moved within the environment, ensuring a reliable visual reference for anthropometric measurements.

### 3.3. Markerless Optical Motion Capture System Proposal

**Figure 1:**
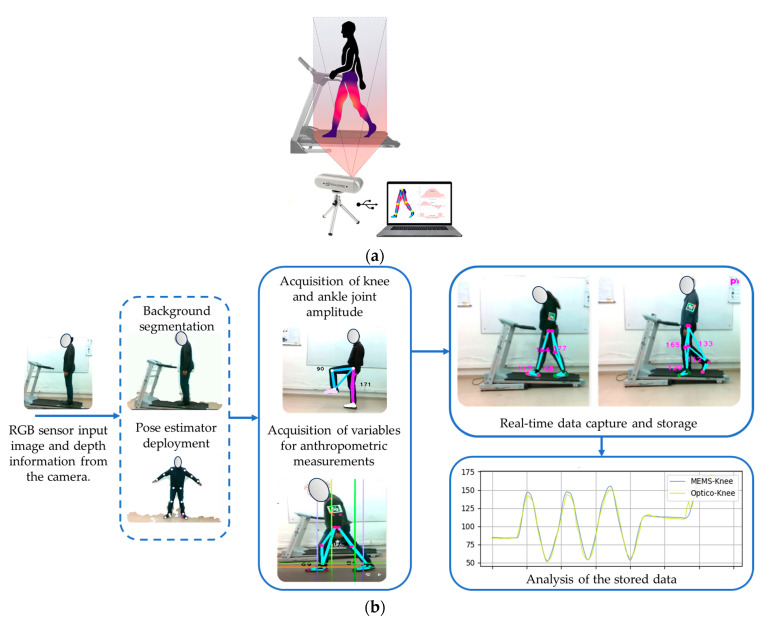
Design and structure of a markerless optical motion capture system for gait analysis and quantification of anthropometric parameters. Part (**a**) shows the structural blueprint of the system. Part (**b**) shows data processing in an algorithmic way.

Figure 1a, presented below, displays the structural blueprint of the system model proposed in this study. It is composed of the Realsense depth camera, providing both RGB and depth channels, and a personal computer running specialized vision algorithms, processing the information captured by the system. The treadmill facilitates the capture of data in the laboratory but can be used without it, indoors and outdoors. Figure 1b shows data processing in an algorithmic way, providing the steps to collect and process data. 

Its key feature is its ease of use, eliminating the need for a meticulously prepared environment. The primary objective of this system is to seamlessly capture anthropometric measurements and identify different states during the gait process. What sets this system apart is the fusion of low-cost optical technologies with advanced machine learning techniques discussed in the previous Materials Section. This combination enables the system to perform real-time biomechanical assessments with unprecedented accuracy and efficiency. In addition, its versatility allows it to be used in a variety of environments, both indoor and outdoor, making it adaptable to different environments and scenarios.

In the context of image and depth processing using the OpenCV and NumPy libraries, it is crucial to emphasize the importance of identifying camera characteristics and parameters. This process serves the fundamental purpose of optimizing the acquisition of information from two primary sources: the RGB sensor, responsible for the acquisition of visual data, and the depth sensor, which complements and enhances the optical acquisition.

**Figure 2:**
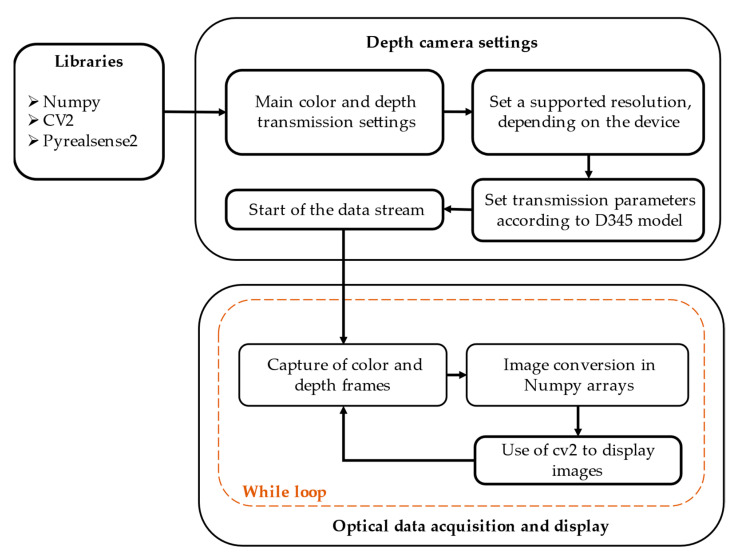
Optical data processing to optimize the capture of color and depth information using open source libraries.

The configuration of the capture environment, which utilizes both visual and depth information, plays a key role in the entire process, as illustrated in Figure 2. This configuration involves a careful consideration of various factors, such as the resolution, focal length, and aperture, among other intrinsic and extrinsic camera parameters. These parameters serve as the basis for calibrating and rectifying images to ensure that they faithfully and accurately represent the scene being captured. Firstly, the main parameters of color and depth transmission are adjusted using contrast balancing in OpenCV to achieve a precise correlation of the captured environment. The depth of field is configured to emphasize the subject’s movements, with a maximum limit of 2 m or dynamically adjusted according to the detection of the moving person. The camera’s working resolution is determined by the processing power of the system and is typically set to 480p at 25 fps. This configuration provides an appropriate balance between visual quality and processing efficiency, which is essential for accurate gait analysis.

In addition, the accurate identification of these parameters is essential for subsequent processes, such as stereo rectification and color and depth image alignment. Such an alignment is of paramount importance in computer vision and 3D perception, facilitating accurate depth perception and spatial understanding in various applications.

In this research, the PyRealSense2 library is used to configure the depth camera according to the specified requirements. In addition, other optical processing libraries are responsible for managing various configurations. These tasks include acquiring device information, handling sensor options, querying and adjusting specific settings, and managing extensions. These libraries also monitor device flow, set trigger states, and read images via device API functions. To acquire and update camera images, a polling function has been implemented to ensure data storage in arrays using NumPy. This approach also allows the camera’s captured information to be displayed through the OpenCV library. A description of the different algorithms that allow data collection and processing of the proposed system is shown in Table 3 below.

**Figure 3:**
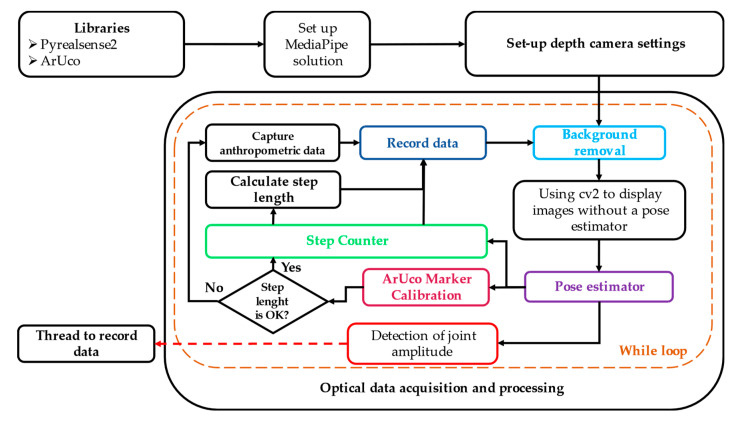
Integration of multiple sensing algorithms for human motion analysis.

Figure 3 illustrates the connection between the key components of the proposal, providing an overview of the implemented architecture and how each element contributes to the analysis of human movement. The step-counting logic and ArUco marker technology are utilized to determine the traveled distance, count the total number of steps, analyze the phases of the gait cycle, and apply a sequential marking method to prevent skipped steps and accurately represent the gait cycles.

## 4. Results

This section presents the outcomes of the evaluation of the proposed system, utilizing the parameters and metrics outlined in the designed test protocol. The aim is to provide a comprehensive performance analysis, shedding light on both strengths and potential weaknesses. Additionally, the system’s performance is compared with established reference systems to offer context and a thorough assessment of its capabilities relative to existing alternatives within the field of study.

### 4.1. Description of the Evaluation and Validation Protocol for the Developed Proposal

This protocol was designed and executed to evaluate the accuracy and reliability of the proposed markerless optical motion capture system. Its primary objective is to verify the system’s capacity to provide pertinent data for applications related to gait analysis and biomechanics, which is vital for its validation and potential optimization. Presented below is a comprehensive examination of the essential constituents of the protocol.

- **Measurement of Anthropometric Values:** This phase involves comparing the anthropometric values obtained from the proposed system, including knee to heel length, hip to ankle length, ankle to toe length, and height, with established measurement standards.
- **Step Length Estimation:** Accurate estimations of each participant’s gait length are conducted to ensure precise gait tracking. The results are then compared to conventional measurements.
- **Joint Amplitude Estimation:** In this stage, the protocol estimates joint angles in the knee and ankle joints of the study subjects and compares them with a commercial MEMS-based reference system.
- **Joint Amplitude Tracking during Gait:** Real-time tracking of the previously estimated joint angles is carried out to analyze movement dynamics. This tracking is then compared with a commercial reference system based on MEMS.
- **Pedometer Verification:** The protocol verifies the proper functioning of the pedometer in accurately recording the number of steps. The results are compared with a manual count.
- **Distance-Traveled Tracking:** Utilizing the step length estimation and pedometer data, the protocol rigorously measures the distance traveled by each participant during the walk against established measurement standards.

### 4.2. Measurement of Anthropometric Parameters

For anthropometric measurements, an ArUco marker is attached to a hook-and-loop strap cuff designed for this purpose. The subject is positioned statically in the sagittal plane, with the marker placed on their left arm. Subsequently, the limb length is measured using a measuring tape to evaluate the accuracy of the system.

The posture estimation and anthropometry detection algorithms are then activated in the system, enabling a precise assessment of the subject’s limb length. The test entails measuring various limb segments of two subjects: from the knee to the heel, from the hip to the left ankle, from the ankle to the toe, and the overall height. These measurements are recorded in centimeters (cms), and the test has a duration of 30 s.

From the values obtained through the proposed system, four samples are taken to calculate the average value and determine the central tendency, taking into consideration any possible skewed distributions or outliers. These calculated values are documented in Table 4.

Table 5 shows the anthropometric data obtained using an optical system and a tape measure. The accuracy of the proposed optical system is evaluated by comparing its mean values with those obtained by the conventional tape measure. This comparison is significant because it serves to validate the trustworthiness of the optical system for collecting anthropometric data and reinforces the credibility of the data collected.

The optical system does show a degree of disparity when compared to measurements obtained with a measuring tape, which indicates the presence of an absolute error. However, it is crucial to emphasize that this error remains at relatively modest levels.

For test subject 1, the maximum error remains well within a tolerance of 5 cm, whereas for test subject 2, the error is approximately 1.2 cm for both height measurements. Furthermore, it is noteworthy that the length measured from hip to ankle exhibits a minimal absolute error, never exceeding 1.03 cm for both individuals. In terms of the relative error, which assesses the precision of the optical system, it generally falls within acceptable margins. However, it is important to highlight that the measurement of the length from the ankle to the sole registers the highest relative error, reaching 1.88% for subject 1 and 7.58% for subject 2. It is crucial to underscore that the assessment of joint angles plays a pivotal role in analyzing and comparing an individual’s posture. These angles furnish essential information about body alignment and stability during physical activities, which is critical for both optimizing the effectiveness of exercise and preventing potential injuries. Therefore, it is imperative to incorporate these measurements to conduct a comprehensive and accurate analysis of the subject’s posture.

### 4.3. Step Length and Traveled-Distance Estimation 

To measure the subject’s step length and traveled distance, three markers were positioned at different intervals on the floor of the measurement lab. The first marker served as the neutral point, offering no data. The second marker was located 30 cm from the reference line, and the third marker was placed 60 cm away from the same reference line. Additionally, an ArUco marker was attached to the subject’s left arm, ensuring it remained in the sagittal plane. For the measurement, the subject’s right foot was positioned initially on the neutral point. In the first sample, the center of the left foot had to align with the second line, while in the second sample, an alignment was required with the third line. The resulting data are compiled in Table 6 below.

It is crucial to consider that the subject’s posture can impact the measurement results. Differences in posture and walking style may affect the accuracy of anthropometric measurements and covered distance, undermining the results of the single-step length estimation. To ensure precise results, it is advisable to maintain the subject in a proper posture during data collection. Moreover, the meticulous calibration of the system parameters can significantly improve the quality of the obtained results.

### 4.4. Estimation of Joint Amplitude

**Figure 4:**
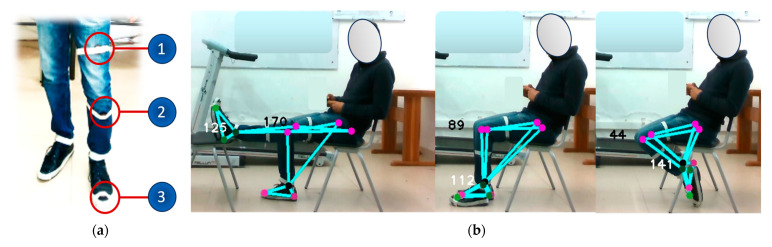
(**a**) Location of MEMS sensors. (**b**) Capture of joint amplitude information by specifying a reference position.

In the joint amplitude estimation phase, the knee and ankle joint angles of the subjects are evaluated. This approach is compared to a commercial system (Movella DOT Sensors) based on MEMS, strategically placed on the thigh, shin, and foot, as shown in Figure 4a. This arrangement of sensors allows the accurate acquisition of movements and joint angles in the lower limbs, allowing the accuracy of the proposed optical system to be evaluated. Similarly, the acquisition of joint amplitude measurements requires the use of a static reference, adopted by means of pre-established postures, as shown in Figure 4b.

Two types of motion capture methods were employed to measure the body angles of the lower limbs: one based on MEMS sensors and the other utilizing the proposed system, which is optical and markerless. After a thorough analysis of the collected data, it was observed that there were no significant differences between the joint angles detected by the two sensors, as demonstrated in Table 7. However, it is worth noting that a relatively higher margin of error was identified in the third posture depicted in Figure 4b.

Despite the presence of absolute errors that reached up to 13°, both types of technologies exhibited their capability to offer accurate and dependable measurements across the majority of the adopted postures. These findings are promising and indicate that the proposed optical system can be considered a reliable option for such measurements.

### 4.5. Monitoring of Joint Amplitude during Gait

In this study, MEMS sensors are used to measure human body motion, specifically at three distinct points: mid-thigh, mid-shin, and mid-foot. Unlike the previous tests, these measurements are conducted while the subject is in motion, rather than in a static position.

#### 4.5.1. General Movement of the Lower Limb

**Figure 5:**
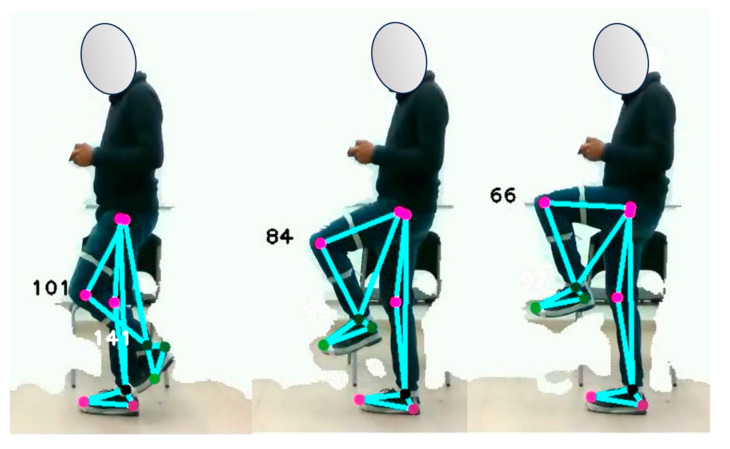
Joint motion of the lower limb of the human body.

The study analyzes the response of the optical system to changes in lower limb motion by jointly examining the knee and ankle angles. Data are collected from MEMS sensors while participants perform three repetitions of leg extension and flexion. The objective is to record the angles, measured in degrees, between the thigh and tibia, as well as between the tibia and instep, while participants are positioned in the sagittal plane in front of the camera. Figure 5 illustrates the details of the typical postures analyzed in the study.

**Figure 6:**
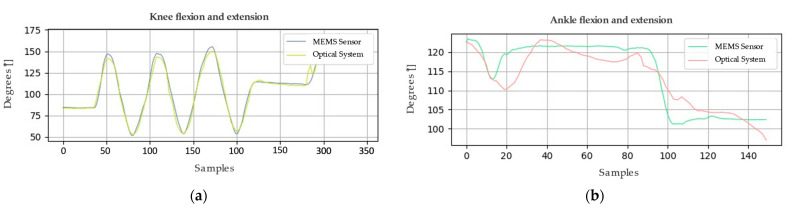
Movement of the lower limb, represented in (**a**) knee and (**b**) ankle joint amplitude values; sample 1.

**Figure 7:**
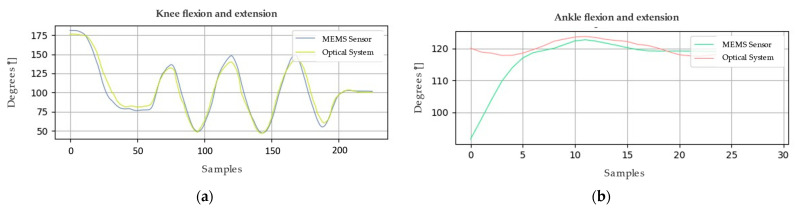
Movement of the lower limb, represented in (**a**) knee and (**b**) ankle joint amplitude values; sample 2.

Similarly, Figure 6 and Figure 7 present the graphical representations of the behavior of the signals obtained from both the optical system and the MEMS sensor array during the analysis of the joint motion of the lower limb. In order to evaluate the accuracy of the system, metrics such as root mean square deviation (RMSD) and root mean square error (RMSE) calculations were employed. These metrics acquire significant relevance in determining the quality of the measurements made by the system.

Concerning the first sample, the RMSE calculations yield crucial results for analyzing the collected data. The RMSE value for the knee is 3.92°, indicating an acceptable level of accuracy. For the ankle, a lower RMSE of 3.63° is recorded, suggesting even higher accuracy. However, when examining the second sample using the same method, the RMSE increases to 6.28° for the knee and 7.29° for the ankle. These elevated RMSE values signify disparities between the actual values and those estimated by the utilized model. It is important to emphasize that these results are pivotal for assessing the model’s accuracy and its ability to predict the values of the variables of interest.

#### 4.5.2. Specific Ankle Joint Movement

**Figure 8:**
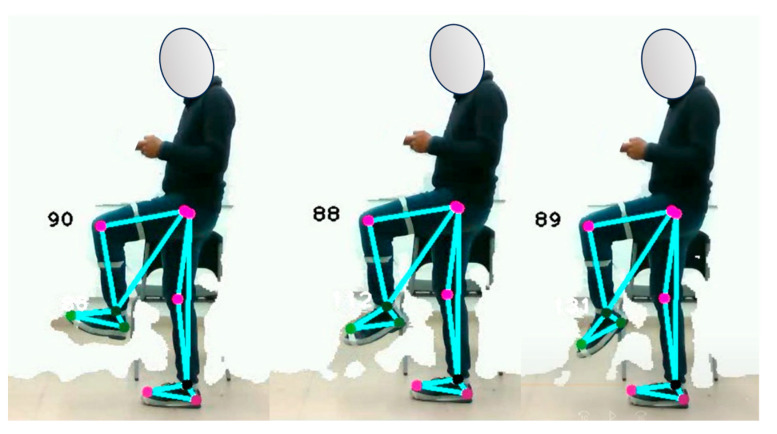
Ankle joint movement, while maintaining a fixed position at the knee joint.

In the study of human motion, the measurement of joint amplitude, especially at the ankle joint, plays a key role. While a fixed range of motion is precisely defined for the knee, the ankle joint mainly involves flexion and extension movements. The detailed test protocol with the detailed measurements is shown meticulously in Figure 8. It is important to emphasize that accurate data collection during this test requires a well-lit environment and accurate positioning of the subject in the plane.

During the execution of this protocol, data were recorded simultaneously from both the proposed optical system and the MEMS sensors. This simultaneous data acquisition allows for in-depth analysis of the signals obtained, facilitating the identification of patterns and trends in ankle behavior.

**Figure 9:**
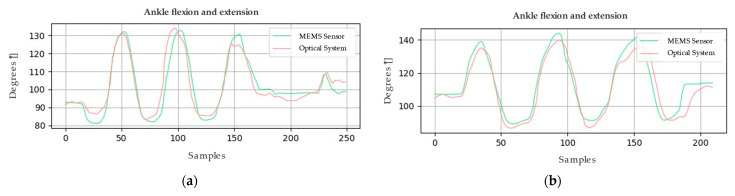
Joint amplitude values for ankle flexion–extension movements. Part (**a**) corresponds to sample 1, while (**b**) to sample 2.

Figure 9 presents the outcomes of the conducted test, wherein both a graphical and mathematical analysis was conducted to compare the response of the optical system with that of the MEMS system under three distinct and noteworthy behaviors. The primary aim of this evaluation was to ascertain the system’s versatility and capacity to adapt to varying circumstances.

The accuracy of the system is assessed through the calculation of the root mean square error (RMSE), yielding a value of 4.71° for both samples examined. This outcome suggests that the system demonstrates a reasonable level of accuracy. Further refinements to the model are recommended to enhance its performance in future experiments. These adjustments might involve selecting novel signal processing algorithms and integrating a broader range of sensors. This can enhance the accuracy and reliability of the system’s results.

#### 4.5.3. Specific Knee Joint Movement

**Figure 10:**
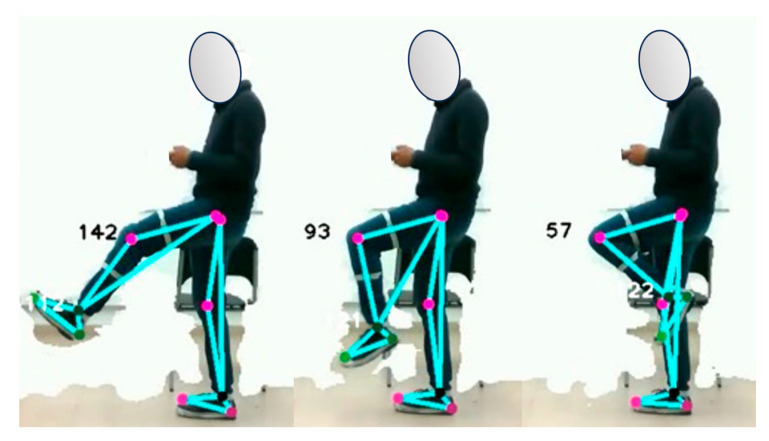
Knee joint motion, while maintaining a fixed position at the ankle joint.

**Figure 11:**
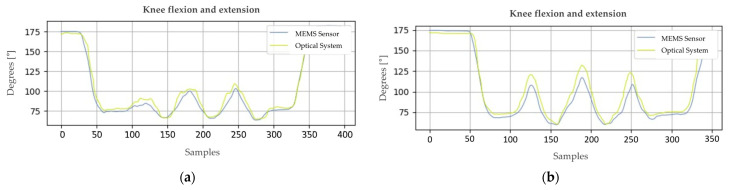
Joint amplitude values for knee flexion–extension movements. Part (**a**) corresponds to sample 1, while (**b**) to sample 2.

The purpose of this test is to characterize how the optical system’s response compares to that of MEMS sensors when measuring knee joint motion. It is imperative that the subject is precisely positioned in the sagittal plane in front of the camera to guarantee the accuracy of the results. A comprehensive outline of the test protocol and the angles measured can be found in Figure 10, while Figure 11 illustrates the outcomes of the conducted test.

Upon conducting a thorough mathematical analysis of the plotted signals, it becomes evident that Figure 11a exhibits an RMSE of 5.46° for the knee angle. A detailed examination suggests that refining the moving average filter layer could enhance the result’s quality, as even minor adjustments can have a substantial impact on result accuracy.

Concerning the data analysis presented in Figure 11b, an RMSE of 9.06° was calculated for the knee angle. While there exists a disparity with the theoretical model, these findings still provide valuable and optimistic insights into the system’s performance. Future experiments may benefit from adjustments to the model or the data collection process to further enhance accuracy.

### 4.6. Verification of the Pedometer

Within the digital pedometer test segment, it is crucial to ensure accurate camera placement to capture the sagittal plane of the subject under study. To perform this task effectively, it is necessary to activate both the pose estimation and the step counter function in the proposed optical motion capture system.

#### 4.6.1. Pedometer Verification at 1.0 km/h

**Figure 12:**
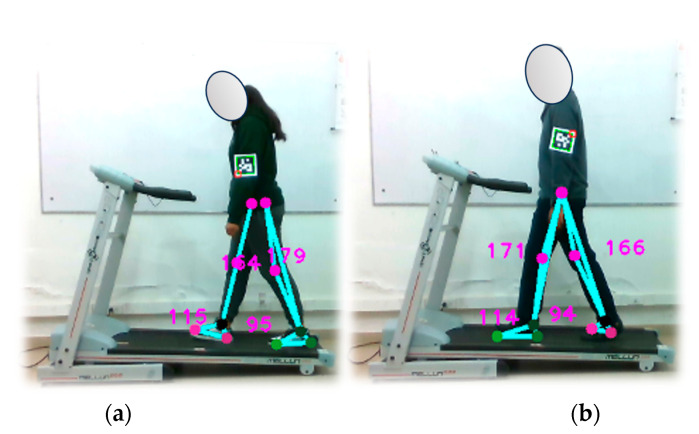
Verification of the pedometer functionality implemented in the optical motion capture system. Walking test at 1.0 km/h. Part (**a**) shows subject 1, while part (**b**) shows subject 2.

In this experiment, the test subject is positioned on an electric treadmill operating at a consistent speed of 1.0 km/h. The optical motion capture system continuously collects samples throughout the walk, as illustrated in Figure 12, capturing the number of steps taken by two individuals. The test is segmented into three categories with step counts of 30, 60, and 90 steps. The outcomes of this experiment are documented in Table 8 presented below.

Throughout the experiment, the subjects were closely observed, with particular attention paid to the potential effect of their clothing on the results obtained. When analyzing the data associated with subject 1, a consistent and highly accurate performance of the optical system was observed. However, when subject 2’s data were examined, significant variability was observed relative to the reference values, resulting in a consistent increase in relative error.

In particular, Figure 12b shows that subject 2 was wearing dark-colored clothing from the waist down. This choice of clothing may have created a color mismatch between the subject’s clothing and the treadmill’s color, potentially contributing to the errors detected by the proposed markerless optical motion capture system. These findings underscore the need for system adjustments to improve its performance in pedestrian stride counting, especially when clothing colors do not provide sufficient contrast between the subject and the environment.

#### 4.6.2. Pedometer Verification at 1.5 km/h

**Figure 13:**
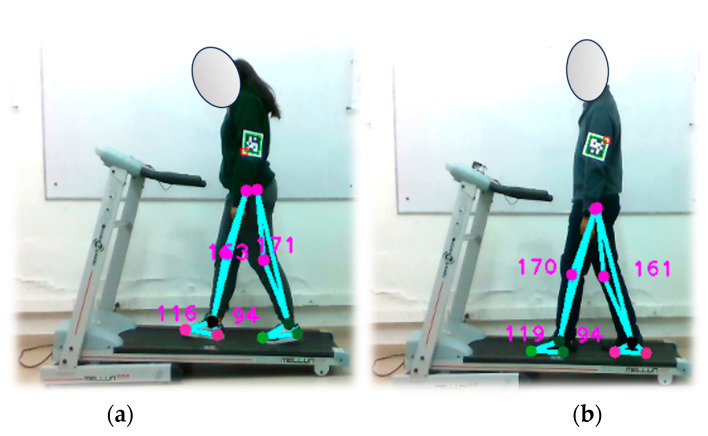
Verification of the pedometer functionality implemented in the optical motion capture system. Walking test at 1.5 km/h. Part (**a**) shows subject 1, while part (**b**) shows subject 2.

A repetition of the previously described test was conducted, but increasing the speed of the electric treadmill to 1.5 km/h. The optical motion capture system continuously sampled data throughout the walk, as depicted in Figure 13. The results of this test can be found in Table 9.

The data clearly reveal that, as the walking speed increases, it places higher demands on the performance of the proposed optical motion capture system, especially in physically more demanding scenarios. Subject 1 demonstrated consistent and accurate step detection by the system, maintaining a good performance. However, for subject 2, there was a noticeable decrease in step detection accuracy compared to the actual steps, indicating that the system may encounter challenges in higher-speed conditions.

These findings underscore the importance of fine-tuning the experimental parameters to enhance the accuracy and effectiveness of the system in detecting footsteps during walking. This optimization process entails refining image processing algorithms, selecting an appropriate sampling rate, and considering factors such as illumination and the attire of the test subjects. As shown in Figure 13, the subjects underwent the same conditions as in the previous test (at 1.0 km/h), suggesting that the pose detection error observed in the case of subject 2 may be a recurring issue. It is crucial to acknowledge the system’s limitations, including its sensitivity to factors like lighting conditions and the clothing combinations worn by the subjects, as these elements can impact the accuracy of the measurements.

### 4.7. Tracking Traveled Distance

To assess the performance of the proposed optical motion capture system concerning the distance covered by the test subject on an electric treadmill, two additional tests were carried out. These tests required the activation of several functions within the motion capture system, including pose estimation, pedometer, and step distance meter, leading to measuring and analyzing the data gathered during the test subject’s walk in both experiments.

#### 4.7.1. Measurement of Traveled Distance at 1.0 km/h

During the testing procedure, the test subject maintains a consistent speed of 1.0 km/h on the treadmill while completing a total of 30, 60, and 90 steps. The assessment of the optical system’s accuracy involves comparing the data supplied by the optical system, expressed in meters, with the distance measured using the markings on the treadmill, spaced 44 cm apart. This approach enables the estimation of the distance covered in relation to the number of steps, and this estimation is then compared with the data generated by the system.

**Figure 14:**
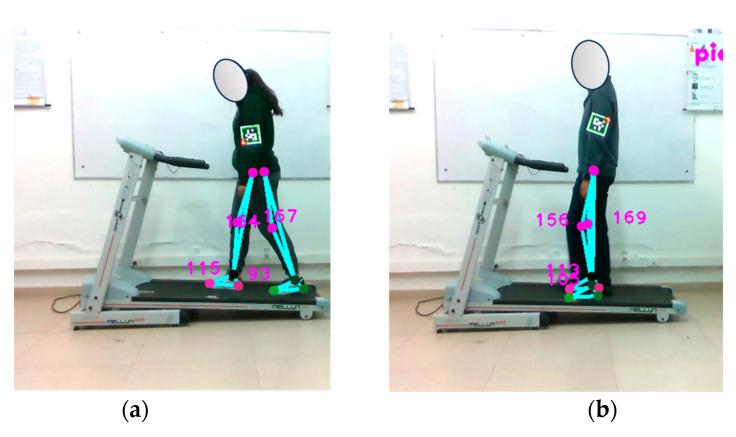
Verification of the distance traveled tracking functionality implemented in the optical motion capture system. Walking test at 1.0 km/h. Part (**a**) shows subject 1, while part (**b**) shows subject 2.

Figure 14 shows the control interface of the optical motion capture system, providing information on the step length and the distance covered by the subject in centimeters. However, it is important to recognize that the position of the ArUco marker in front of the camera may fluctuate during body movement, which could potentially impact the accuracy of data in future measurements. All the collected data are meticulously recorded in Table 10.

Upon completion of the tests, the absolute error of the collected data was determined and compared with the exact values to evaluate the accuracy of the proposed system. The results show that, in general, the optical system provides accurate results with a relative error of less than 11%. 

However, it was found that the absolute error increases with distance, indicating the importance of considering this variable when measuring over long distances. It is important to note that, despite this limitation, the data obtained using the treadmill are closer to the exact value, which supports the reliability of the proposed system. Consequently, these results represent an additional validation of the proposed method to obtain accurate measurements in motion tests, thus strengthening its potential use in future studies.

#### 4.7.2. Measurement of Traveled Distance at 1.5 km/h

**Figure 15:**
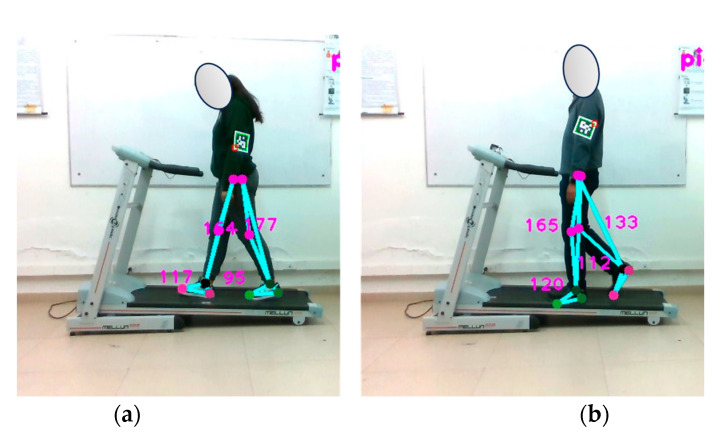
Verification of the distance-traveled tracking functionality implemented in the optical motion capture system. Walking test at 1.5 km/h. Part (**a**) shows subject 1, while part (**b**) shows subject 2.

The test procedure requires the subject to maintain a constant speed of 1.5 km/h on the treadmill and to complete a total of 30, 60, and 90 steps. The experiment is shown in Figure 15, while all the data are shown in Table 11.

Each person has a unique, repetitive walking pattern that aims to minimize effort and energy expenditure, resulting in varying stride lengths between individuals. Figure 15 illustrates this, showing that subject 1 has a stride of 41.88 cm while subject 2 has a stride of 45.36 cm. Notably, subject 1 exhibited a significant increase in error during the test. Several factors, such as stride length, can influence the captured data. Stride length can vary not only between different individuals, but also for the same individual under different conditions. Consequently, it is essential to consider these limitations when interpreting results and assessing the system’s performance in diverse situations.

### 4.8. Measurement of Gait Phases

The objective of the test is to assess the performance of an optical motion capture system in analyzing and segmenting the different stages of the bipodal gait cycle. The gait cycle comprises several distinct phases, including:

- **Heel strike:** When the foot initially touches the ground.
- **Foot flat:** Involving a shift in body weight.
- **Midstance:** When the foot bears the most weight.
- **Heel off:** Marking the start of the leg’s movement away from the ground.
- **Initial swing:** Propelling the body forward.
- **Mid swing:** As the leg advances for the next step.

These phases work together in a coordinated manner to enable efficient and balanced walking. The test’s primary goal is to evaluate how accurately and reliably the system captures these phases.

**Figure 16:**
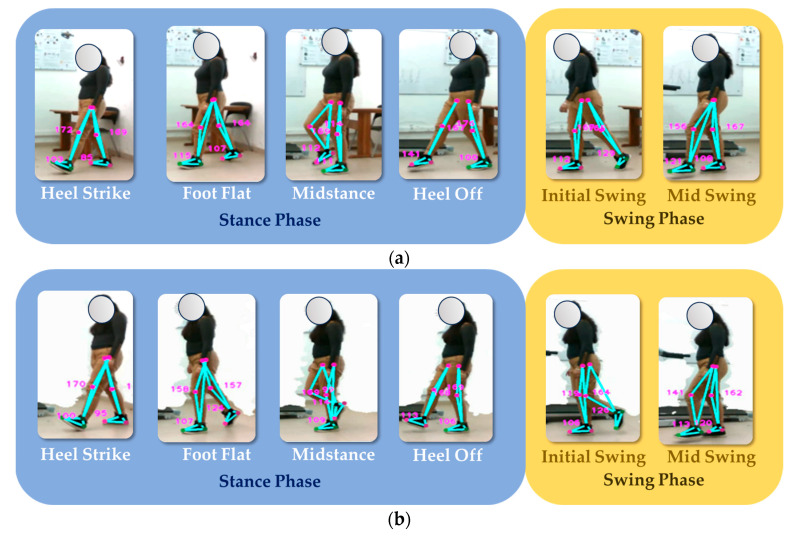
Segmentation and identification of the sequence of gait phases by means of the proposed optical motion capture system. Analysis of test subject 1. Part (**a**) corresponds to sample 1, while part (**b**) corresponds to sample 2.

To perform this evaluation, the subject must walk at an average speed for several gait cycles to allow the depth camera to accurately record the sagittal plane. Two gait cycles are performed, as shown in Figure 16. Accurate data acquisition is essential for gait analysis and the assessment of gait-related conditions. It is recommended that the subject wear tight-fitting clothing during the test to minimize data errors caused by loose clothing or external disturbances.

**Figure 17:**
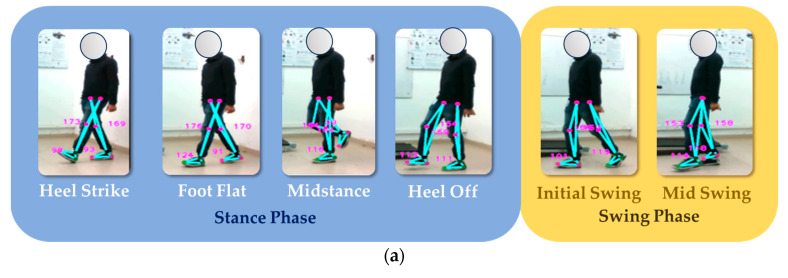

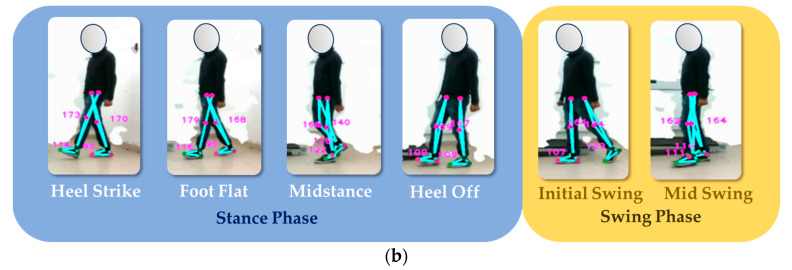
Segmentation and identification of the sequence of gait phases by means of the proposed optical motion capture system. Analysis of test subject 2. Part (**a**) corresponds to sample 1, while part (**b**) corresponds to sample 2.

The analysis of Figure 16 shows that the body positions during the various gait phases are consistent across the samples and are consistent with positions documented in the literature. While there is a slight difference in the angle of the ankle of the right leg during the heel-off phase between the two samples, it is noteworthy that the system correctly identified this phase. In addition, the distinction between the midstance phase and the descent phase serves as a valuable indicator for assessing the subject’s balance. For further insight, see Figure 17, which illustrates the experiment conducted with the second subject.

The experiment with the second subject shows that, during the heel strike phase, a knee joint angle of more than 168° is generated when touching the ground. In the midstance phase, the ankle angle of the stance foot is between 122° and 116°, with a difference of only 6°. During the stance phase, the images show that the foot has not yet touched the ground, which is an important finding according to the literature. During the transition from the deceleration phase to the mid-swing phase, a pronounced forward motion is observed, triggering the generation of momentum for the next gait cycle.

The study has shown that the proposed optical motion capture system can segment and identify the different stages of human gait, as summarized in Table 12. This achievement is made possible by establishing a relationship between foot movements and phase changes, which allows the development of an efficient algorithm for identifying these stages.

The application of the developed system in rehabilitation and movement assessment has significant potential benefits. It can be of great use in detecting pathologies early and in monitoring the recovery of patients with locomotor system injuries. The ability to automatically identify different phases of gait may allow healthcare professionals to design personalized rehabilitation plans and monitor patient progression over time. In addition, this tool can be valuable for evaluating the efficacy of treatments and for research in the field of gait biomechanics.

## 5. Discussion and Conclusions

The importance of human movement research in various fields, including sports, healthcare, industry, and entertainment, is now widely recognized. For this reason, this study focuses on the design, implementation, and testing of a low-cost, portable optical motion capture system. To accomplish this, the prototype was built around a depth-enabled RGB camera, and various gait analysis functions were created utilizing the open-source machine learning solution offered by MediaPipe. These functions have been augmented with references to ArUco markers, streamlining the process of acquiring anthropometric values in an efficient and convenient manner.

The intelligent algorithms integrated in this prototype are capable of identifying a person and generating a set of key points to represent the human silhouette or identify specific features, as mentioned in other studies [6,7,47,48]. The absence of physical markers provides users with a streamlined experience, eliminating the need for additional accessories or uncomfortable adjustments. In addition, this feature enhances the mobility and flexibility of the system, making it a highly practical and effective tool for assessing various health conditions and analyzing gait in an unobtrusive manner [1,49,50].

The prototype underwent testing in controlled laboratory conditions to meticulously assess its reliability and performance. To establish a benchmark for comparison, MEMS sensors, specifically Movella DOT sensors, were employed. This comprehensive comparative analysis offers profound insights into the effectiveness and potential of the proposed optical system, highlighting its notable advantages, such as portability, affordability, and precision, in both anthropometry and gait phase analyses. MEMS sensors are widely recognized for their ability to deliver precise real-time measurements, proving indispensable in various medical research and diagnostic applications. Furthermore, MEMS technology adeptly addresses challenges associated with visual occlusion, ensuring the reliability of measurements, even in complex scenarios [38,51,52]. Thus, they serve as invaluable tools for validating the performance of the proposed prototype. The parameters obtained through this system hold immense promise for evaluating neurological diseases, neuromotor disorders, and monitoring patients’ health, as underscored in prior research [9,14,19,47].

The testing protocol designed to evaluate the proposed system under a laboratory setting is divided into several stages, focusing on different aspects of gait analysis and biomechanics. These stages include the measurement of anthropometric values, estimation of step length, estimation of knee and ankle joint amplitude, real-time tracking of joint angles, pedometer verification, and tracking of distance traveled. The assessment protocol stands out for its accurate measurement of anthropometric values, such as knee to heel length, hip to ankle length, ankle to toe length, and height. This protocol serves as a tool for managing various system functions, with a particular focus on the ability to assess real-time responses. By employing various evaluation metrics in the protocol, it becomes possible to objectively assess the platform’s usability, identify areas for potential improvement, and make informed decisions to optimize the platform and enhance efficiency.

The results obtained from the tests conducted as part of the protocol are satisfactory, indicating that the system operates within the specified design parameters. The highest relative error in the anthropometric parameter measurement tests does not exceed 7.6%, and the maximum absolute error is 4.67 cm at an average height. Additionally, the maximum relative error in stride length measurement was 11.2%. Regarding the static and dynamic joint angle tests, the maximum average errors, in terms of percentage, were found to be 10.2% and 9.06%, respectively. These findings indicate that the system is capable of furnishing anthropometric data and joint angles with a commendable level of accuracy. While these results establish its viability as a valuable tool for real-time biomechanical evaluation, it is worth noting that future investigations will aim to validate its clinical utility for applications, such as rehabilitation environments and medical diagnostics assistance.

## 6. Future Work

As part of future work, the integration of additional technologies, such as Doppler-based sensors, is proposed to address potential occlusion issues that may arise in machine learning solutions. This integration could also provide supplementary data to enhance measurement accuracy. Furthermore, the implementation of threading and parallelization in specific project areas is suggested to significantly boost system performance, both in terms of processing speed and data quality.

In addition to the aforementioned suggestions, another potential improvement lies in exploring alternative pattern recognition algorithms. Current systems rely on specific algorithms that may have limitations in detecting certain movements or physical features. By integrating multiple algorithms, the system can achieve more precise and efficient pattern recognition, ultimately improving the quality of the collected data.

To further improve system performance, it is recommended to introduce dedicated accelerators, such as GPUs or TPUs. These accelerators can process large amounts of data more quickly and efficiently than traditional CPUs. Their integration would allow the system to process real-time information faster and more accurately, resulting in higher-quality data and an improved user experience.

Finally, through the utilization of multiple cameras instead of a single depth camera, it is believed that the accuracy of the system can be further improved. The use of multiple cameras allows the capture of data from different angles and perspectives, enabling more accurate measurements of angles and motion. This approach also mitigates occlusion issues and generally enhances data quality.

## Figures and Tables

**Table 1:** Description of the research methodology, composed of four independent stages.

N°	Stage Description
1	Summary of the state of the art in motion sensing technologies.
2	Development of a lower limb motion capture system utilizing a depth camera and MEMS sensor.
3	Creation and implementation of an assessment and validation protocol for the implemented technological setup, enabling a comparative analysis between optical and MEMS technologies.
4	Evaluation and synthesis of findings obtained through the validation protocol.

**Table 2:** Materials used in the proposal’s implementation.

Material	Category	Use
Intel D415 Stereo Depth Camera	Hardware (H)	Collection of movement and depth information.
NumPy and Pandas libraries	Software (S)	Numerical processing of the information, e.g., dataset elaboration.
MediaPipe and Pyrealsense2 libraries	Software (S)	Graphic processing libraries, including pose detection, joint segments, and speed, among other parameters.
OpenCV and ArUco libraries	Software (S)	Complementary graphic processing libraries.

**Table 3:** Description of the implemented algorithms.

N°	General Description	Algorithm Operation
**1**	Acquisition of knee and ankle joint angles	The information from the depth camera is used to measure the angles of the knee and ankle joints. Key points are located to establish a coordinate system (x, y) covering the entire lower limb. Using mathematical methods, values corresponding to the position and orientation of the joints are calculated.
**2**	Data storage using threads	A thread is utilized to continuously record the data while the program is running. The generated dataset undergoes processing and filtering to obtain a more stable signal, reducing fluctuations and noise.
**3**	Capture of gait information	The gait analysis focuses on the values of joint amplitudes and step counts as key variables. This component provides an accurate and systematic method for measuring human motion, including the identification of the heel and toe moments and their corresponding values.
**4**	Acquisition of anthropometric measurements	The ArUco module allows for the detection and localization of a marker. This information is valuable for subsequently obtaining various anthropometric measurements, where a ratio between the marker’s size in pixels and its actual size in centimeters is applied.
**5**	Capture of step length	This technique is employed to calculate the user’s traveled distance. It measures the length from the middle of the right foot in relation to the middle of the left foot. These measurements are recorded and processed to obtain the final value, representing the total traveling distance.
**6**	Detection of gait phases	Conditions for recognizing the phases in the gait cycle are established using a sequential flag approach to prevent step skipping and ensure accuracy. The gait phase detection relies on the components described above.

**Table 4:** Anthropometric measurements taken with the proposed system.

Item	Samples from Subject 1				Samples from Subject 2			
	1	2	3	4	1	2	3	4
Knee to heel length [cm]	46.53	45.60	45.88	46.94	47.66	47.96	48.73	48.29
Hip to ankle length [cm]	91.21	90.19	90.31	92.39	93.98	93.02	92.70	91.93
Ankle to toe length [cm]	25.68	25.61	25.50	26.47	24.63	22.97	23.18	23.51
Height [cm]	171.02	169.10	169.34	173.23	176.21	174.41	173.81	172.37

**Table 5:** Anthropometric measurements on a measuring tape and average of the values taken from the optical system.

Subject	Measurement Type	Knee to Heel Length	Hip to Ankle Length	Ankle to Toe Length	Height
**Subject 1**	Optical System [cm]	46.24	91.02	25.82	170.67
	Measuring Tape [cm]	45.20	90.00	24.00	166.00
	Absolute Error [cm]	1.04	1.02	1.82	4.67
	Relative Error [%]	2.30	1.14	7.58	2.81
**Subject 2**	Optical System [cm]	48.16	92.91	23.55	174.20
	Measuring Tape [cm]	48.00	93.00	24.00	173.00
	Absolute Error [cm]	0.16	0.09	0.45	1.20
	Relative Error [%]	0.33	0.09	1.88	0.69

**Table 6:** Summary of step length measurements in relation to floor marks.

Measurement Using Optical System	Subject 1		Subject 2	
	30 cm	60 cm	30 cm	60 cm
**Sample 1 [cm]**	32.90	59.34	29.72	58.11
**Sample 2 [cm]**	34.95	59.85	29.36	58.24
**Sample 3 [cm]**	32.21	59.92	30.05	57.56
**Mean [cm]**	33.35	59.70	29.86	57.97
**Absolute Error [cm]**	3.35	0.35	0.11	2.03
**Relative Error [%]**	11.16	0.50	0.36	3.38

**Table 7:** Summary of joint amplitude measurement data by specifying a reference position.

Measurement	Subject 1						Subject 2					
	Thigh and Shin			Tap and Foot			Thigh and Shin			Tap and Foot		
**Reference [°]**	180	90	45	120	110	130	180	90	45	125	110	130
**Optical System [°]**	170	89	44	132	109	138	175	89	44	125	112	141
**MEMS Sensor [°]**	168	95	47	123	110	130	169	96	43	121	109	128
**Absolute Error [°]**	2	6	3	9	1	8	6	7	1	4	3	13
**Relative Error [%]**	1.1	6.3	6.4	7.3	0.9	6.1	3.6	7.3	2.3	3.3	2.8	10.2

**Table 8:** Digital pedometer test results at 1.0 km/h.

Measurement	Subject 1			Subject 2		
	30 Steps	60 Steps	90 Steps	30 Steps	60 Steps	90 Steps
**Optical System**	30	60	90	32	72	114
**Absolute Error**	0	0	0	2	12	24
**Relative Error [%]**	0	0	0	6.67	20.00	26.67

**Table 9:** Digital pedometer test results at 1.5 km/h.

Measurement	Subject 1			Subject 2		
	30 Steps	60 Steps	90 Pasos	30 Steps	60 Steps	90 Pasos
**Optical System**	30	60	90	28	54	70
**Absolute Error**	0	0	0	2	6	20
**Relative Error [%]**	0	0	0	6.67	10.00	22.20

**Table 10:** Results of the 1.0 km/h trip-distance tracking test.

Measurement	Subject 1			Subject 2		
	30 Steps	60 Steps	90 Steps	30 Steps	60 Steps	90 Steps
**Optical System [m]**	13.83	26.72	41.14	12.92	28.67	44.99
**Treadmill [m]**	13.20	26.40	39.60	14.08	31.70	50.16
**Absolute Error**	0.63	0.32	1.54	1.16	3.03	5.17
**Relative Error [%]**	4.77	1.21	3.88	8.24	9.60	10.30

**Table 11:** Results of the 1.5 km/h trip-distance tracking test.

Measurement	Subject 1			Subject 2		
	30 Steps	60 Steps	90 Steps	30 Steps	60 Steps	90 Steps
**Optical System [m]**	12.41	24.65	35.98	11.87	21.84	27.66
**Treadmill [m]**	13.20	26.40	39.60	12.32	23.76	30.80
**Absolute Error**	0.79	1.75	3.62	0.45	1.92	3.14
**Relative Error [%]**	5.98	6.63	9.14	3.65	8.08	10.19

**Table 12:** Summary of the identification of gait phases using the proposed optical system.

Gait Phase		Subject 1		Subject 2	
		Sample 1	Sample 2	Sample 1	Sample 2
**Stance Phase**	Heel strike	✔	✔	✔	✔
	Foot flat	✔	✔	✔	✔
	Midstance	✔	✔	✔	✔
	Heel off	✔	✔	✔	✔
**Swing Phase**	Initial swing	✔	✔	✔	✔
	Mid swing	✔	✔	✔	✔

## Data Availability

The original contributions presented in the study are included in the article, further inquiries can be directed to the corresponding authors.
